# Threshold effect of growth rate on population variability of *Escherichia coli* cell lengths

**DOI:** 10.1098/rsos.160417

**Published:** 2017-02-22

**Authors:** Manasi S. Gangan, Chaitanya A. Athale

**Affiliations:** Division of Biology, Indian Institute of Science Education and Research (IISER) Pune, Dr Homi Bhabha Road, Pashan, Pune 411008, India

**Keywords:** cell size, bacteria, multi-fork replication, population variability, RecA, bacterial cell division

## Abstract

A long-standing question in biology is the effect of growth on cell size. Here, we estimate the effect of *Escherichia coli* growth rate (*r*) on population cell size distributions by estimating the coefficient of variation of cell lengths (CV_L_) from image analysis of fixed cells in DIC microscopy. We find that the CV_L_ is constant at growth rates less than one division per hour, whereas above this threshold, CV_L_ increases with an increase in the growth rate. We hypothesize that stochastic inhibition of cell division owing to replication stalling by a RecA-dependent mechanism, combined with the growth rate threshold of multi-fork replication (according to Cooper and Helmstetter), could form the basis of such a threshold effect. We proceed to test our hypothesis by increasing the frequency of stochastic stalling of replication forks with hydroxyurea (HU) treatment and find that cell length variability increases only when the growth rate exceeds this threshold. The population effect is also reproduced in single-cell studies using agar-pad cultures and ‘mother machine’-based experiments to achieve synchrony. To test the role of RecA, critical for the repair of stalled replication forks, we examine the CV_L_ of *E. coli ΔrecA* cells. We find cell length variability in the mutant to be greater than wild-type, a phenotype that is rescued by plasmid-based RecA expression. Additionally, we find that RecA-GFP protein recruitment to nucleoids is more frequent at growth rates exceeding the growth rate threshold and is further enhanced on HU treatment. Thus, we find growth rates greater than a threshold result in increased *E. coli* cell lengths in the population, and this effect is, at least in part, mediated by RecA recruitment to the nucleoid and stochastic inhibition of division.

## 1. Introduction

The size and shape of a cell is considered a characteristic feature of a given cell type, and quantifying its variability in a population provides information about the effect of fluctuations on a complex phenotype. *Escherichia coli* cells have typically been described as spherocylinders of length 2 µm and width 1 µm. Differences in sizes are primarily owing to cell length (*L*) and not so much the width [1–3]. Cell length frequency distributions show a positive skew owing to the presence of long cells (*L* > 8 μm), the proportion of which is increased by environmental factors such as low bacterial density at 22°C and 37°C or a shift to richer media [4]. In *Salmonella*, growth rate (*r*) alone has been shown to correlate with increased cell size and multiple nucleoids [5], but a microscopic study on *E. coli* has shown that cells grown at 22°C are shorter than at 37°C [4]. The effect of temperature and growth medium on cell size appears thus to suggest that growth rate might primarily regulate the cell size. However, the quantitative relationship and molecular mechanism by which growth could affect cell sizes remains unclear.

The growth rate of bacteria, in particular, *E. coli,* is regulated by numerous pathways that typically connect growth to nutrient availability [6–8]. Many genetic factors that link nutrient sensing to cell size regulation have been identified [9–11]. These pathways, however, link growth rate via pathways independent of replication to cell size. If DNA replication fails to complete and the bacterial nucleoid does not segregate, the nucleoid ‘occlusion’ response results in cell elongation [12–14]. Based on the BCD—birth (B), chromosome replication (C) and division (D)---cycle [15], growth rates exceeding one doubling per hour (doubling time, *t*_d_ = 60 min) result in insufficient time for the completion of the chromosome replication (C-period approx. 40 min) and cell division (D-period approx. 20 min). Cooper and Helmstetter postulated and experimentally demonstrated that *E. coli* undergoes simultaneous rounds of replication, multi-fork replication [16] to overcome the shortening of *t*_d_ in rapid growth. However, the role of multi-fork replication in cell size regulation has not yet been investigated.

Recent improvements in light microscopy image analysis have allowed quantification of bacterial morphology and growth dynamics with subpixel accuracy [17–19]. Combined with fluorescence microscopy of subcellular components [20,21], it has become possible to address single-cell dynamics of the bacterial cell division cycle. These advances now allow us to address the effect of population sizes and physical factors and probe the mechanisms that control cell sizes and cell size variability.

Theoretical studies have suggested that asymmetric cell division [2], lognormal distribution of growth rates [22] or stochastic partitioning of molecular components at cell division [23] could lead to a heterogeneity in cell sizes in the population. Recent single-cell bacterial growth kinetic data [19] combined with theoretical modelling have reopened the debate of whether cell-size robustness is determined by a ‘timer’ or ‘sizer’ mechanism [24] and currently the ‘incremental’ or ‘adder’ model appears to explain all available data [25,26]. However, the effect of molecular regulatory networks on cell size and the correlation of cell size variability with growth rate remain unclear.

RecA is a central regulator of the SOS response pathway, and deletion mutants of *E. coli* for the *recA* gene experience enhanced replication fork stalling [27]. Additionally, a *recA1* mutation is known to result in asynchronous replication and a reduction in the expected genome-copy numbers [15]. In previous work, we had found that a *recA1* mutation phenocopies typical cell septation defects, resulting in elongated cells containing multiple nucleoids and increased cell length variability [28]. While replication fork stalling and repair are important for DNA replication, as reviewed by Cox *et al*. [29], the artificial induction of replication stalling results in increased cell lengths [30]. The repair of stalled replication forks by RecA protein assembly on DNA [31] also triggers SulA-mediated cell division inhibition [32] via the SOS response pathway [33–35]. At the same time, the population growth rate affects the number of replication forks per cell in a step-wise manner [16]. As a result, the number of replication stalling events could be multiplicatively increased by growth rate and thus affect cell division. Therefore, we hypothesize that RecA might provide the molecular link between *E. coli* growth rate and cell length.

Here, we measure the correlation between cell length variability and growth rate from steady-state cultures, and test our method against single-cell agar-pad and microfluidic growth assays. We find that cell size variability remains unchanged for slow-growing cultures, but increases above a threshold growth rate. By increasing replication fork stalling with hydroxyurea (HU) in multiple mutant strains, we demonstrate that DNA replication fork dynamics can affect population cell size distributions in a RecA-dependent manner. From the growth-rate-dependent recruitment of RecA to the genome, we infer a molecular mechanism that links growth rate to cell size.

## 2. Material and methods

### 2.1. Bacterial strains and plasmids

Multiple *E. coli* strains were used: MG1655 (6300, CGSC), ΔrecA (JW26691, CGSC), ΔsulA (JW09411, CGSC), ΔslmA (JW56411, CGSC) and *E. coli* MG1655 with a GFP-tagged genomic copy of *recA* (*recA-GFP*) was grown in the presence of 25 µg ml^−1^ kanamycin as described previously [36] (gift from Dr G.P. Manjunath). Nucleoid segregation dynamics were followed in *E. coli* MG1655 with a pBAD24-hupA-gfp plasmid with 100 µg ml^−1^ ampicillin [37] (gift from Dr Josette Rouviere-Yaniv). We constructed two *recA* expression plasmids (i) *mCherry* tagged and (ii) arabinose-inducible, untagged. Two primer sets were used with complementary regions to the genomic RecA sequence and overhangs for restriction digestion for the p-recA-mCherry and pBAD-recA constructs (electronic supplementary material, table S1). The *recA* gene was PCR-amplified (Mastercycler proS, Eppendorf, Germany) using Taq polymerase and dNTPs (Bangalore GeNei, India) in recommended buffers. The template DNA, *E. coli* MG1655 genomic DNA, was extracted by a rapid extraction method that avoids polysaccharide contamination [38]. The *recA* amplicon for mCherry tagging and the p-mCherry plasmid were sequentially digested with *Sal*I and *Hind*III. The fragments were separated on an agarose gel, column-purified (QIAquick, Qiagen, Germany) and ligated using a T4 DNA Ligase (Bangalore GeNei, India). The plasmid p-mCherry was constructed by replacing the GFP sequence in a pGFP plasmid with mCherry from p-mCherry-N1 (both plasmids from Clontech, USA) by directional cloning using the restriction enzymes *Sal*I and *Eco*RI. The *recA* amplicon for arabinose-inducible expression was purified, and both the amplicon and pBAD24 digested sequentially by *Nhe*I and *Xba*I, and ligated as before. Plasmids were transformed using the CaCl_2_ method [39] in *E. coli* DH5α cells. Plasmids were isolated using a spin column-based method (Miniprep Kit, Qiagen GmbH, Germany).

### 2.2. Growth media

For rapid growth, cells were grown in Luria–Bertani (LB) broth (HiMedia, Mumbai, India), while reduced growth rate was achieved using the reduced media yeast extract broth (YEB): 0.5% (w/v) yeast extract in 1% (w/v) solutions of NaCl and tryptone broth (TB): 1% (w/v) tryptone in a 1% (w/v) solution of NaCl. Additionally, M9 minimal salts medium [40] supplemented with 4 µg ml^−1^ thymidine were reconstituted with three different carbon sources (to result in successively slower growth rates): 0.4% (w/v) glucose or 0.9% (w/v) succinic acid or 0.5% (w/v) sodium acetate (all sugars from Sigma-Aldrich). All broths and media were made in deionized water and the pH was adjusted to 7.

### 2.3. Batch culture and growth rate estimation

Cells were grown at 37°C with shaking at 180 r.p.m. (Forma, ThermoScientific, USA) in 100 ml LB, YEB and TB using a 1% overnight inoculum. Identical conditions were used to grow *E. coli* MG1655 in M9 + sugars. Cell density was estimated by converting 1 OD_600 nm_ = 8 × 10^8^ cells ml^−1^ [41]. To estimate the growth rate (*r*), the averaged OD with time curves were fit to the solution to the logistic equation by

2.1 $$N(t)=\frac{N(0)\cdot K}{N(0)+(K-N(0))\cdot {\text{e}}^{-r\cdot t}},$$

where *N*(0) is the population at the time of inoculation, *r* is the growth rate (h^−1^), *K* is the carrying capacity and *t* is time (electronic supplementary material, figure S1). Doubling time is *t*_d_ = 1/*r* [42].

### 2.4. Continuous cell culture

A PDMS-based microfluidic device was used to grow *E. coli* MG1655 culture continuously based on the ‘mother machine’ design [19]. The device was designed as a two-layered micro-pattern mask in CleWin (WieWin Web, The Netherlands) and fabricated by using an approximately 100 nm layer of gold (for aligning the second layer) followed by spin-coating a 2 µm layer SU8-2 negative photoresist (Microchem, USA) onto a SiO_2_ wafer using a spin coater model WS-400B-6NPP LITE (Laurell Tech. Corp., USA). The photoresist was cured by UV exposure with a mask (EVG, Austria) corresponding to the trench and dead-end channels. Unexposed photoresist was washed and a 20 µm layer of SU8-20 negative photoresist (Microchem, USA) spun and exposed to UV corresponding only to the trench, for curing. The PDMS device was made by mixing elastomer : curing agent of 10 : 1 w/w (Sylgard 184, Dow-Corning, USA) and coating the wafer and heat-curing it in an oven at 60°C (Raut Scientific, Maharashtra, India) for 2 h. The cured PDMS membrane was cleaned with pentane (Sigma-Aldrich, Mumbai, India) and washed with acetone (Fisher Scientific, Mumbai, India). The air-dried device was then bonded in air using a plasma cleaner (Emitech K050X, Quorum Technologies, UK) under RF power of 70 W, washing time of 30 s and 1 mbar vacuum (Edwards Pumps, UK). The PDMS-glass device was integrated and channels passivated by passing 10 mg ml^−1^ of BSA (Sigma-Aldrich, Mumbai, India) for 1 h. *E. coli* MG1655 cells were infused into the device (OD ∼ 1.0) and allowed to diffuse into the channels for 1 h at 37°C using a syringe pump (PHD Ultra, Harvard Apparatus, USA). The device was washed by flowing in either fresh LB medium or M9 + succinate at a constant flow rate of 0.3 ml h^−1^. Cell growth and division were observed in DIC microscopy. To measure the effect of HU treatment in continuous culture, *E. coli* MG1655 and Δ*recA* strains expressing eGFP from a plasmid were introduced in the microfluidics device as before. Cells were grown under continuous flow in LB for 1 h (approx. three generations), followed by a change of the medium to the corresponding medium supplemented with 30 mM HU for 1 h (‘treatment’). Subsequently, the medium without any drug (no HU) was once again replaced for 2.5 h of ‘recovery’. Fluorescence time-lapse images were acquired in the GFP channel and analysed.

### 2.5. Hydroxyurea and trimethoprim treatment

Overnight cultures were grown from a single colony of *E. coli* MG1655, Δ*recA*, Δ*sulA*, Δ*slmA* and Δ*recA* + pRecA-mCherry. The cultures were diluted 1 : 100 (1% inoculum) into 5 ml of fresh LB and M9 + 0.9% succinate and grown at 37°C with shaking (180 r.p.m.). *E. coli* MG1655 with genomic RecA-GFP was similarly grown in LB, TB and YEB at 37°C with shaking. At OD_600 nm _∼ 0.2, the cultures were incubated in 10–100 mM HU containing growth medium for three generations corresponding to 1 h in LB, 1.5 h in TB, 2 h in YEB, 3 h in M9 + succinate. Subsequently, cells were allowed to recover for another three generations. Similarly, *E. coli* MG1655 cells grown in LB and M9 + succinate were exposed to 1 µg ml^−1^ trimethoprim (Sigma-Aldrich, India) and allowed to recover for 1 and 3 h, respectively. After recovery, all cultures (treated and untreated) were washed, fixed and imaged.

### 2.6. Western blotting

*Escherichia coli* MG1655 and *ΔrecA* cells grown in LB, YEB and TB media were grown for three generations and treated with HU as above. The OD at 600 nm was measured before treatment and after recovery, and 1 ml cell suspensions were diluted to result in comparable cell densities (electronic supplementary material, table S2). The cells were pelleted, washed in phosphate-buffered saline (PBS), resuspended in 50 µl of lysis buffer consisting of 10 µl of 5× SDS loading dye (250 mM Tris–Cl (pH 6.8), 10% SDS, 50% glycerol, 0.5% bromophenol blue and 500 mM DTT) and 40 µl PBS, heated to 95°C for 10 min with constant shaking at 700 r.p.m. (ThermoMixer, Eppendorf, Germany), and samples were centrifuged before loading on a 10% SDS–PAGE gel run at 120 V (Bio-Rad, USA). Proteins were transferred onto a PVDF membrane (Immobilon-P transfer membrane, EMD Millipore Corporation, USA) and the membrane blocked with 5% milk powder in TBST buffer (Tris–Cl-buffered saline (pH 7.4) and 0.1% Tween 20). Rabbit antiserum raised against *E. coli* RecA [43] (a gift from Dr K. Muniyappa) was diluted to 1 : 12 000 in blocking agent (5% milk powder in TBST buffer) and incubated with 100 ml of cell lysate of *E. coli* Δ*recA* for 12 h at 4°C to immunodeplete non-specific antibodies. The lysate was prepared by growing *E. coli* Δ*recA* cells to OD_600 nm_ approximately 2.0, resuspending the pellet in PBST (PBS with 0.05% (v/v) Tween 20) and lysis by pulse sonication for 2 min, 30 cycles. The membrane was incubated with this pre-treated serum at 4°C overnight, washed and hybridized with the secondary HRP-conjugated anti-rabbit antibody, 1 : 10 000 diluted (Jackson ImmunoResearch, USA). The blot was developed using a reagent Luminata Femto (Millipore Corporation, USA) and luminescence images acquired (LAS 4000, GE Healthcare, USA).

### 2.7. Cell immunostaining

*Escherichia coli* MG1655 were grown to the mid-log phase and cells fixed with 1.6% paraformaldehyde (PFA) and 0.01% glutaraldehyde, incubated for 1 h and washed three times with PBST. The cells were treated with GTE (50 mM glucose, 25 mM Tris–Cl, 10 mM EDTA) containing 5 µg ml^−1^ lysozyme and incubated at 37°C for 45 min, followed by 3× wash with PBST. The cell suspension was spread on poly-l-lysine-coated coverslips and air-dried for 1 h. Coverslips were washed three times with PBST, incubated with 2% BSA (blocking agent) for 1 h, washed with PBST and incubated with the rabbit anti-RecA serum (1 : 1000 diluted in PBS) at 4°C for approximately 12 h. The coverslip was then incubated at 37°C for 1 h with Alexa647 conjugated anti-rabbit antibody (Thermo Fisher Scientific, USA) and mounted on slides.

### 2.8. Fluorimetry

*E. coli recA-GFP* was grown in 5 ml of LB, TB and YEB (1% inoculum) with 25 µg ml^−1^ kanamycin at 37°C with constant shaking. The cultures were treated with 30 mM HU as above. Cell suspensions (1 ml) were sampled at pre-treatment (pt) and recovery (r) stages, and r-samples were diluted in the respective growth medium based on the ratio of OD recovery : pre-treatment samples (LB: pt 0.23, r 1.3; YEB: pt 0.195, r 0.92; TB: pt 0.193, r 0.819). All samples were pelleted and resuspended in 50 µl PBS and fluorescence measured in a 96-well half-area round bottom black plate (Corning, USA) using 480 nm excitation and 510 nm emission in a Varioskan Flash multifunctional plate reader (Thermo Scientific, USA). Measurements were blank-subtracted by measuring *E. coli* MG1655 of the same density grown in LB, YEB and TB. Fluorescence per cell was estimated by dividing by the total cell numbers in 50 µl by using the conversion 1 OD_600 nm_ = 8 × 10^8^ cells ml^−1^ as before.

### 2.9. Microscopy

Cells were sampled (200 µl) from the batch cultures at the mid-log phase and fixed in 4% PFA, stained with 0.1 µg µl^−1^ of DAPI (Sigma-Aldrich, India) and mounted, as has been described previously [28]. Fixed cells of *E.coli* MG1655 *recA-GFP* and *E. coli* Δ*recA* expressing RecA-Cherry were acquired using the 100× (Plan Apochromat N.A. 1.4, oil) objective of a Zeiss Axio Imager Z1 (Carl Zeiss, Germany) microscope in fluorescence and DIC channels. For live-imaging, cells were grown on 2% agar pads with 100 µg ml^−1^ ampicillin and induced for 2 h by 0.2% Arabinose (Sisco Research Labs, Mumbai, India) to express HupA-GFP and imaged on a Zeiss LSM780 confocal microscope (Carl Zeiss, Germany) simultaneously in fluorescence (Diode laser 405 nm, beam splitter MBS 405, pinhole 126.5 corresponding to 1 airy unit) and DIC modes using a 63× lens (Plan Apochromat NA 1.40, oil). Multiple positions were scanned as 512 × 512 pixel images (0.264 µm per pixel) with an image acquired every 2 min for approximately 2 h and at 37°C.

### 2.10. Image analysis

Cell lengths were automatically analysed from DIC images of fixed cells using a previously developed algorithm [28] in Matlab R2014b (MathWorks Inc., MA, USA). The source code has been released on a GPL basis and can be downloaded from a Github repository (https://github.com/athale/ecolilenDIC). The birth lengths and the division lengths of *E. coli* cells in the ‘mother machine’ and RecA puncta were interactively estimated using ImageJ (v. 1.50f) [44]. Kymographs of *E. coli* cells expressing plasmid-based eGFP grown in the ‘mother machine’ were generated by using ‘multiplekymograph’ plugin in ImageJ [44] based on a line of interest drawn along the growth channel. RecA puncta were quantified by selecting a segmented line corresponding to the length of the *E. coli* cell in DIC and used to generate an intensity profile in the RecA-GFP and DAPI channels. Co-localized peaks were used to score cells in the population and calculate the percentage cells showing such co-localization of RecA on the nucleoid. To follow the dynamics of nucleoid segregation, intensity profiles from timeseries of *pHupA-GFP*-transformed cells were plotted as a matrix to produce a kymograph (space–time plot), using the *imagesc* function in Matlab R2014b (MathWorks Inc.). Western blot intensity analysis was performed using ‘gel analyser’, an ImageJ plugin. The protein band area was obtained by using the ‘label peaks’ function and maximum normalized for comparison.

### 2.11. Data analysis

Cell frequency distributions were normalized by the sum of the area under the curve, fit to a lognormal distribution to obtain lognormal mean (*µ*) and variance (*v*) using *fitdist*, *lognpdf* and *lognstat* functions using Matlab with the Statistics Toolbox (MathWorks Inc.). The Kolmogorov–Smirnov (KS) test statistic was calculated for the number of bins (*n* = 44) and significance level (*α*) of 0.01 to arrive at a test statistic (*D*_(α, n)_) given by [45,46]

2.2 $$D_{\left(\alpha ,n\right)}=\frac{\sqrt{-\ln(\alpha \text{/}2)}}{2n}.$$

The cumulative distribution function (CDF) of observed and fit data was calculated for each bin (i) from the length–frequency distribution. The difference $\left|d_{i}|=|F_{i}-{\hat{F}}_{i}\right|$ between the observed (*F_i_*) and expected (${\hat{F}}_{i}$) values of the CDF was evaluated, and the maximum (*d*_max_) was found. The hypothesis that the fit to the data was good was accepted if *d*_max_ < *D*(*α,n*). Variability in cell lengths was quantified by the coefficient of variation (CV_L_) using the expression CV_L_ = *σ*_L_/*μ*_L_, where *σ*_L_ and *μ*_L_ are the standard deviation and mean of cell lengths, respectively.

## 3. Results

### 3.1. Growth rate affects population cell length distributions of *Escherichia coli* MG1655

**Figure 1.**
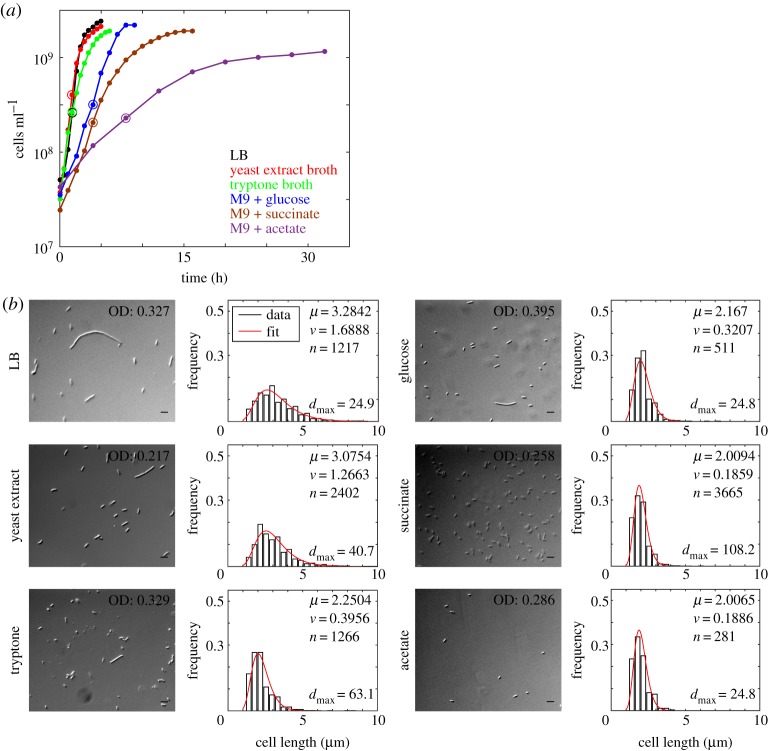
Growth rate and cell size distributions. (*a*) The growth (log_10_ cell density) of *E. coli* MG1655 cells was measured as a function of time in the following growth media: LB (black), yeast extract broth (YEB) (red), tryptone broth (TB) (green), M9 media supplemented either with glucose (blue), succinate (brown) or acetate (purple). Samples taken from the mid-log phase (circles) (*b*) (left) were examined in DIC microscopy (scale bar, 5 µm) and (right) the cell length distribution (bars) plotted. The distribution was fit to lognormal (red) where *μ*: mean cell length, *v*, variance; *n*, total number of cells. *d*_max_ is the Kolmogorov–Smirnov test value evaluated for the fit.

With the aim of measuring the effect of growth rate, *r*, on cell size, *E. coli* MG1655 cells were grown in LB, YEB, TB and M9 supplemented with glucose, succinate and acetate. As expected, the growth of cells was the fastest in LB and decreased for all other media with the slowest growth observed in M9 supplemented with acetate (figure 1*a*). Doubling time (*t*_d_) values were obtained from logistic function (equation (2.1)) fit to the growth curves (electronic supplementary material, figure S1) and ranged between 33 and 273 min (table 1). Cells sampled from the mid-log phase of each culture (figure 1*a*) were imaged, analysed and the frequency distributions of cell lengths fit to a lognormal function (figure 1*b*). The goodness of the fit was validated based on the KS non-parametric test (electronic supplementary material, table S3). The number of cells analysed in each sample ranged between 10^2^ and 10^3^ cells, comparable to previous microscopic studies on population cell size distributions [2,47]. Corresponding to the decrease in growth rate, the cell length distributions also decreased in spread. The spread of the distribution was maximal in samples grown in LB and minimal in M9 + acetate, with intermediate growth rates (in YEB, TB, M9 + glucose, M9 + succinate), resulting in an intermediate spread of cell lengths. The growth rate thus appears to alter the quantitative nature of the cell length distribution, while leaving the qualitative nature (lognormal) unchanged. Dynamic imaging of a population of *E. coli* MG1655 cells expressing HupA-GFP to label the DNA demonstrated that most cells divided normally, whereas elongated cells arose rarely and were accompanied by hampered DNA segregation (electronic supplementary material, video S1). This hints at cell division failure and DNA replication–segregation coupling as a potential cause for the observed variability of cell lengths. However, because the population distributions analysed from fixed cell microscopy are taken from unsynchronized bulk cultures, we proceeded to examine if the cell-cycle stage does indeed affect our analysis, using live cells in continuous culture.

**Table 1.** The doubling times and growth rates of *E. coli* MG1655 were estimated from fitting the logistic equation to average (*n* = 3) OD measurements with time. Cultures were grown at 37°C with constant shaking.

growth medium	doubling time, *t*_d_ (min)	growth rate, *r* (h^−1^)
LB	33.11	1.814
yeast extract broth	39.87	1.505
tryptone broth	57.2	1.049
M9 + 0.4% glucose	63.13	0.9504
M9 + 0.9% succinate	131.98	0.4546
M9 + 0.5% acetate	273.35	0.2195

### 3.2. Single-cell analysis of lengths of newborn and dividing cells in microfluidics

**Figure 2.**
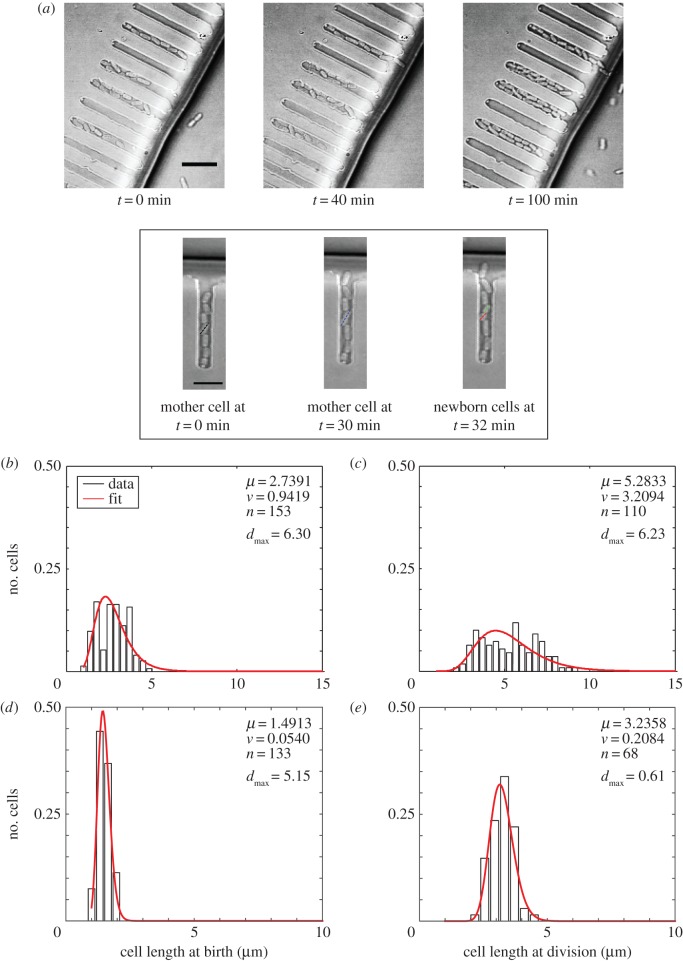
Cell length distribution of wild-type *E. coli* in continuous culture. (*a*) Representative DIC images of *E. coli* MG1655 cells grown in continuous culture in a ‘mother machine’ device with LB were recorded at 0, 40 and 100 min (also electronic supplementary material, video S3). Scale bar, 10 µm. Inset: a representative channel is marked to indicate a mother cell (black line), which grows in 30 min (blue line) and divides into two daughter cells at 32 min (green and red lines). The cell length distributions of cells grown in two growth media: (*b,c*) LB and (*d,e*) M9 + succinate were measured at birth (*b,d*) and division (*c,e*) and the frequency distributions are fit by lognormal distributions (red) with parameters *µ* and *v* and the goodness of fit was measured by *d*_max_ (evaluated for the KS test, electronic supplementary material, table S1).

The ‘mother machine’ microfluidics device described previously by Wang *et al*. [19] is ideally suited for single-cell analysis of rod-shaped cell growth dynamics. We capture birth and division events and estimate cell lengths (figure 2*a*) from timeseries of cells grown in LB (electronic supplementary material, video S2) and M9 + succinate (electronic supplementary material, video S3) at 37°C. The frequency distribution of the cell lengths from single-cell analysis also fit a lognormal distribution (figure 2*b–e*), similar to the fixed-cell data, with the goodness of fit validated by the KS Test (electronic supplementary material, table S3). While the variance of cell lengths in LB showed a difference between newborn cells (figure 2*b*) and cells just prior to division (figure 2*c*), the mean cell length of newborn cells was also smaller (*μ* = 2.7 µm, *v* = 0.9 µm^2^) than dividing cells (*μ* = 5.28 µm, *v* = 3.29 µm^2^). As a result, the normalized variability measured by the coefficient of variation of cell lengths (CV_L_) remained constant for cells grown in LB---0.3188 for newborn cells (arithmetic mean 2.73 µm, s.d. 0.87 µm) and 0.3129 for dividing cells (arithmetic mean 5.27 µm, s.d. 1.65 µm). This suggests that population cell length variability is independent of cell growth stage, based on the two extreme cases, i.e. newborn and dividing cells in the same growth medium. Compared with LB, cell length distributions of cultures grown in M9 + succinate have a narrower spread in data from both newborn (figure 2*d*) and dividing (figure 2*e*) cells. The CV_L_ of these cells is 0.156 (newborn) and 0.139 (dividing), twofold smaller than those measured in LB, confirming the qualitative impression. This suggests that the cell length variability as measured by CV_L_ from single-cell experiments is independent of the cell-cycle stage in a given medium, while changing growth rates lead to measurable differences in the CV_L_. To validate this finding, we also examine the growth of microcolonies, which form natural populations.

### 3.3. Growth-rate dependence of cell size variability in microcolonies

**Figure 3.**
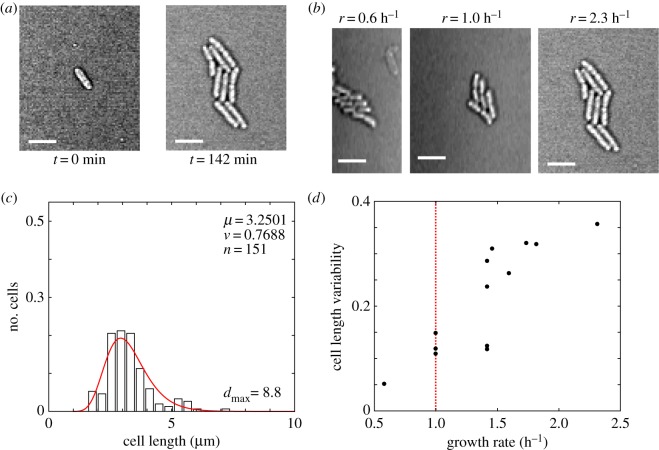
Population cell size variability in a microcolony. (*a*) The growth from a single cell to a microcolony at the end of 140 min is depicted. (*b*) Multiple microcolonies originating from a single cell after 140 min of growth are of different sizes, indicating differences in growth rates. Scale bar is 5 µm. (*c*) The cell length frequency distribution (bar) pooled from 13 microcolonies is fit by a lognormal distribution (red). *μ*, mean; *v*, variance; *n*, number of cells analysed, *d*_max_, the KS test statistic measure. (*d*) The variability of cell lengths in a microcolony measured by the CV is plotted as a function of the average microcolony growth rate (h^−1^). The dotted vertical line indicates a growth rate of *r*_mf_ = 1 h^−1^.

Agar-pad-based single-cell dynamics are routinely used to examine cell division dynamics in *E. coli*. We followed the growth dynamics of single cells, as they formed microcolonies at 37°C on LB agar for 140 min (figure 3*a*; electronic supplementary material, video S4). Consistent with previous reports of growth rate heterogeneity in single cells [48], we find that growth rates vary in the range of 0.6–2.3 h^−1^ in the population (figure 3*b*). Each colony examined originates from a single cell, and hence at the end of 140 min when microcolony sizes vary, the cell–cell variation in growth rates is confirmed. The population cell length distribution of these microcolonies also fit a lognormal function (figure 3*c*), with the goodness of fit evaluated using the KS test (electronic supplementary material, table S3). To our surprise, the CV_L_ from individual microcolonies appeared to increase with increasing growth rate (figure 3*d*). Because the sample size in each CV_L_ measurement of a single microcolony is very small, and the growth-rate difference between single cells is difficult to control and is possibly the result of intrinsic stochastic variability, we instead proceeded to modulate average growth rate by the nutrient medium and analyse cell size variability in fixed cell microscopy, to take advantage of better population statistics.

### 3.4. Cell length variability affected by a combination of growth rate, *recA* and hydroxyurea

**Figure 4.**
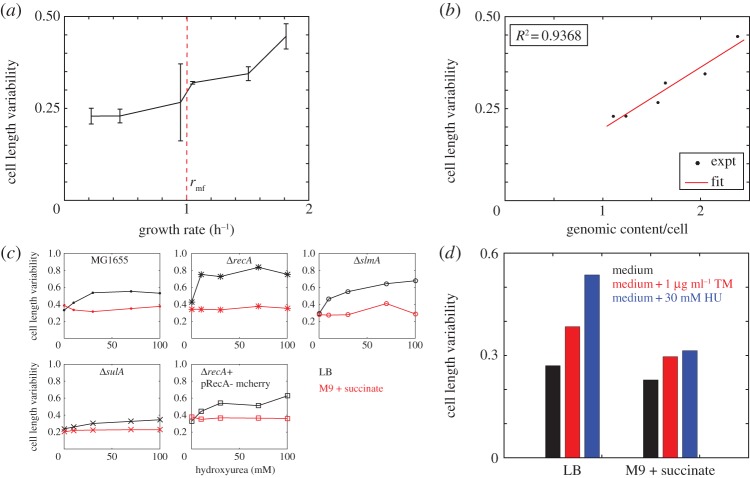
Effect of growth rate and replication stochasticity on cell lengths. (*a*) The cell length variability from the mid-log phase of cultures (*y*-axis) is plotted as a function of the growth rate (*x*-axis). The growth rate of *r*_mf_ = 1 h^−1^ (red line) is the multi-fork replication threshold based on Cooper & Helmstetter [16]. Error bars indicate s.d. (*b*) The measured cell length variability (*y*-axis) is plotted as a function of the expected total cellular DNA (*x*-axis) based on the model of Zaritsky *et al*. [49] (equation (3.1)). (*c*) Cell length variability of *E. coli* (*y*-axis) with increasing hydroxyurea (HU) concentration (*x*-axis) was estimated for the following strains: MG1655 (dots), Δ*recA* (asterisks), Δ*slmA* (circles), Δ*sulA* (x) and Δ*recA* + pRecA-mCherry (open squares). Cultures were grown either in LB (black) or M9 + succinate (red). (*d*) The cell length variability of *E. coli* MG1655 populations (*y*-axis) grown in LB or M9 + succinate (black) and compared with cells grown in the same medium but treated with 1 µg ml^−1^ of trimethoprim (TM) (red) or 30 mM HU (blue).

Cell length variability was quantified in the mid-log phase of cells grown in media resulting in growth rates ranging between 0.2 and 1.81 h^−1^ by using LB, the reduced media YEB, TB and M9 supplemented with sugars, to modulate growth rates (table 1). We find that cells grown in M9 supplemented with glucose, succinate and acetate are less variable (CV_L_ < 0.25), and the variability increases gradually with increasing growth rate (figure 4*a*). When the growth rate (*r*) exceeds 1 division per hour (in TB, YEB and LB), the CV_L_ appears to enter a second phase of a steeper increase. This inflection point also correlates with the growth rate threshold for multi-fork (mf) replication (*r*_mf_ = 1 h^−1^) [16]. Because multi-fork replication changes the genomic content per cell (*G*), we used a previously developed expression relating *G* with the BCD cycle [49] and doubling times, to estimate it as

3.1 $$G=\frac{t_{d}\cdot (2^{(C+D)/t_{d}}-2^{D/t_{d}})}{C\cdot \ln2},$$

where *t*_d_ is the doubling time, *C* is the period of chromosome (DNA) replication and *D* is the time for cell division (septum formation). We combine the experimentally measured doubling times for different growth media (table 1) with an assumed *C*-period of 40 min and *D*-period of 20 min [16,50]. We find that the measured CV_L_ is positively correlated to increasing values of the estimated average genome content (*G*) per cell (figure 4*b*). This correlation demonstrates that genome content and cell size regulation could be coupled.

Because the genome content dependence of CV_L_ does not, however, reproduce a biphasic, threshold dependence in cell length variability (figure 4*a*), we examined whether perturbing replication dynamics below and above the presumptive growth rate threshold could be used as a test of replication stochasticity as the underlying mechanism. HU is known to induce stochastic replication fork stalling [51,52] and the RecA protein is critical for restarting stalled replication forks [31]. Expectedly, cells mutant for *recA* have a reduced ability to recover stalled replication forks [35]. To our surprise, on treatment with HU, both wild-type and Δ*recA* cells showed an increase in CV_L_ when grown in LB, but not when grown in M9 + succinate (figure 4*c* and electronic supplementary material, figure S5). Thus, slow growth appears to protect cells from the cell-division defects of HU, but rapid growth induces an increase followed by saturation in cell length variability. *E. coli* Δ*recA* mutants, however, differ from wild-type, because the difference of CV_L_ between LB and M9 + succinate was more pronounced. On the other hand, minimal medium-grown cells lacking *sulA*, ‘the effector’ of RecA, are less variable when compared with wild-type and do not respond to HU treatment. The treatment of LB and minimal medium-grown Δ*slmA* and Δ*recA* cells expressing RecA-mCherry cells results in variability comparable to MG1655. The ‘rescue’ of the wild-type phenotype by expression of RecA in a mutant background and the variability of Δ*sulA* mutant suggest the specificity of the RecA-SulA mechanism for growth-rate-dependent regulation of cell size. Additionally, the increase and saturation of cell size variability on treatment with HU of wild-type cells only during rapid but not slow growth further validate the threshold growth rate dependence of cell length variability. The growth-rate-dependent increase in cell length variability with trimethoprim treatment (figure 4*d*), a drug known to increase replication fork stalling [53], reinforces replication stochasticity as a mechanism regulating cell size. Based on this, we hypothesize that, in addition to its previously known roles, RecA recruitment to replication fork stalls [54] could mechanistically relate growth rate with cell size variability.

### 3.5. Single-cell dynamics of *Escherichia coli* MG1655 and *ΔrecA* with hydroxyurea treatment

**Figure 5.**
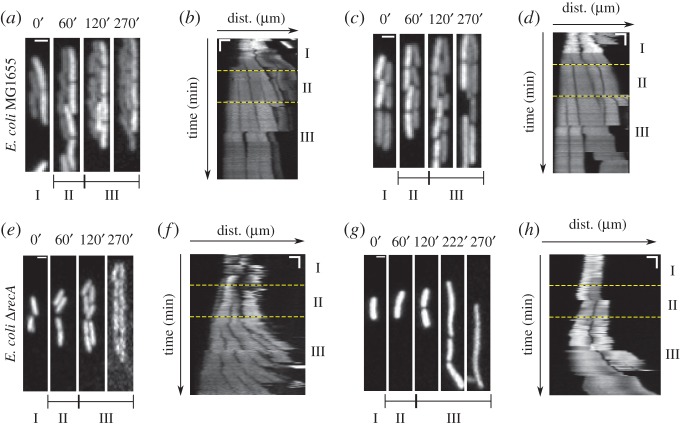
Effect of hydroxyurea (HU) on *E. coli* strain expressing eGFP grown in the ‘mother machine’. (*a,c*) Images of *E. coli* MG1655 and (*e,g*) *E. coli* Δ*recA* mutants were both grown at 37°C in the ‘mother machine’ with three phases in the nutrient supplied I: LB (‘pre-treatment’), II: LB + 30 mM HU (‘treatment’) and III: LB (‘recovery’). Scale bar is 2 µm. (*b,d,f,h*) Kymographs of the preceding timeseries are used to follow cell division. The yellow horizontal lines mark the three phases. Time is in minutes. Horizontal scale bar is 3 µm and the vertical scale bar is 25 min.

Based on this evidence from fixed cells, we expect that HU treatment should affect single-cell dynamics in a manner similar to the effect at a population level. To test this, *E. coli* MG1655 cells expressing eGFP from a plasmid were grown in the ‘mother machine’ in LB with the medium changed in three stages: 1 h pre-treatment (stage I), 1 h 30 mM HU treatment (stage II) and 2.5 h recovery from HU (stage III). Most cells continued to divide normally (figure 5*a*) and a few appeared to undergo moderate filamentation (cell lengths approx. 7 µm) at the end of the ‘recovery’ period (figure 5*c*). The kymographs suggest that the division of some cells is unaffected (figure 5*b*), while a few undergo filamentation (figure 5*d*). Most *E. coli ΔrecA* cells continued to divide normally after recovery from treatment (figure 5*e*), whereas others became prominently filamentous, resulting in cell lengths of approx. 12 µm (figure 5*g*). The kymographs confirmed regular divisions of most cells (figure 5*f*), while a failure of division in some resulted in cell filamentation (figure 5*h*). This qualitatively corroborates our observations from population measurements that (i) cell division failure results in elongated cells, (ii) cell filamentation is probabilistic and (iii) the extent of cell filamentation is greater in *ΔrecA* cells owing to a more extreme filamentation phenotype, when compared with *E. coli* MG1655. From population and single-cell dynamics, we hypothesize that cells that rapidly divide and are treated with HU are expected to have a higher frequency of filamentation and greater variability owing to increased RecA recruitment to the DNA. We proceed to test this hypothesis using microscopy.

### 3.6. Nucleoid localization of recA corresponds to increased cell length variability

**Figure 6.**
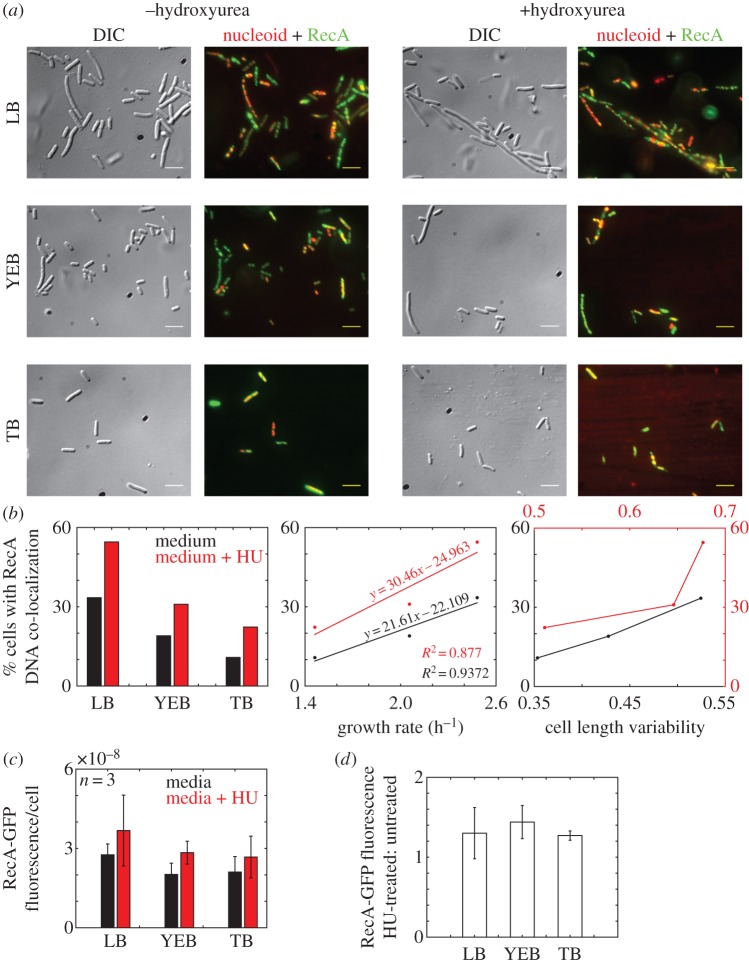
Growth rate dependence of RecA co-localization with nucleoids. (*a*) Mid-log cultures of *E. coli* expressing RecA-GFP (genomic) grown in LB, YEB and TB with (+) and without (−) 30 mM hydroxyurea (HU) were imaged in DIC (grey), and the fluorescence. The merged image of DAPI (red) and GFP (green) fluorescence with co-localization (yellow) is shown. Scale bar, 5 µm. (*b*) The proportion of *E. coli* cells in which RecA foci co-localize with nucleoids (*y*-axis) is plotted against three growth media (*x*-axis) with (red) and without (black) HU, growth rate (h^−1^) (*x*-axis) and cell length variability (*x*-axis). (*c*) Mean (±s.d. from three samples) RecA-GFP fluorescence/cell from fluorimetry of liquid cultures is plotted as a bar chart for untreated (black) and HU-treated (red) cells grown in three media: LB, YEB and TB. (*d*) The ratio of RecA-GFP fluorescence of HU treated to untreated cells grown in LB, YEB and TB are compared.

To test the hypothesis of growth rate-dependent recruitment of RecA to the genome, an *E. coli* MG1655 strain expressing an endogenous RecA-GFP protein [36] was grown in three different media: LB, YEB and TB, to modulate growth rates as before. RecA foci co-localization with the nucleoid (labelled with DAPI) appeared to increase when cells were grown in LB when compared with YEB and TB (figure 6*a*). Treatment with HU resulted in increased co-localization when compared with untreated cells grown in the same growth medium (figure 6*a*). The proportion of cells with co-co-localization of the RecA protein on the nucleoid is low (less than 15% cells with RecA-DNA co-localization) during slow growth (TB), intermediate (approx. 15%) for cells in YEB and high (approx. 30%) in LB during rapid growth (figure 6*b*). The co-localization percentages are further increased on HU treatment. This is evidenced by the percentage cells with co-localized RecA-GFP and DNA increasing linearly with growth rate for untreated cells, with a 1.4-fold increase in the slope for HU-treated cells (figure 6*b*). The percentage co-localization of treated and untreated cells correlates positively with CV_L_, consistent with our previous results of increasing cell length variability with growth rate and HU treatment. As before, the variability of HU-treated cells is higher for the same growth medium, when compared with untreated cells. To test if the co-localization of RecA with the nucleoid was not the result of an artefact of GFP tagging of RecA, the native RecA protein was immunostained in fixed cells of *E. coli* MG1655 and analysed for co-localization with DNA by DAPI staining in multiple fields of view (electronic supplementary material, figure S6*a*). The percentage cells with RecA co-localized on the nucleoid matched the values from the RecA-GFP co-localization (electronic supplementary material, figure S6*b*). In addition, the measured cell length variability from the DIC images was also comparable between the RecA-GFP-expressing strain and the immunostained samples (electronic supplementary material, figure S6*c*). Alternatively, we also imaged the localization of RecA-mCherry protein expressed from a plasmid in *E. coli ΔrecA* cells grown in LB (electronic supplementary material, figure S7*a*) and found the RecA-nucleoid co-localization to be comparable to anti-RecA and RecA-GFP-based quantification (electronic supplementary material, figure S7*b*). The CV_L_ of all three samples remained comparable (electronic supplementary material, figure S7*c*), suggesting that RecA protein tagging did not result in artefacts in either localization or cell length variability. To test if the increased co-localization could have resulted from RecA protein abundance instead of recruitment, we measured the RecA-GFP concentration per cell in fluorimetry and found that HU-treated cells had a slightly increased (approx. 1.25-fold) RecA-GFP expression per cell, for all three growth media tested (figure 6*c*). Western blotting using anti-RecA antiserum of cells grown in LB, YEB and TB with and without treatment also appear to show increased protein in cell lysates on HU treatment, independent of the growth medium (electronic supplementary material, figure S8*a*). Quantification showed an approximately 1.5- to threefold increase in RecA (electronic supplementary material, figure S8*b*) and RecA-GFP intensity after HU treatment, in all growth media tested, not owing to loading artefacts (electronic supplementary material, figure S8*c,d*). Additionally, the expression of RecA-GFP from the genomic locus also increased by 2.5-fold on HU treatment (electronic supplementary material, figure S8*e,f*), with non-specific protein content remaining comparable (electronic supplementary material, figure S8*g,h*). Taken together, the results suggest that RecA recruitment to the nucleoid could serve as one of multiple mechanisms that drive growth rate-dependent cell size variability in clonal populations, independent of RecA protein abundance.

## 4. Discussion

While the study of average values of cell size [55,56] and single-cell studies [19,26,48] have demonstrated that cell size is robust to environmental changes, understanding the population distribution remains important to the ecology of microbes and their survival in changing environments [57,58].

Here, we have examined the population variability of clonal cell sizes and their link to growth rate. We demonstrate that at a fixed growth rate, the cell length variability is constant and independent of the cell-cycle stage using a microfluidics-based continuous culture system. Additionally, the growth rate of microcolonies on an agar pad appears to correlate with cell-cycle synchronized variability in cell lengths. However, in our analysis, quantitative fixed cell microscopy from bulk cultures results in better statistics and a more robust control over growth rates. We find increasing growth rates increases the population variability in cell lengths in a bi-phasic manner, with the two phases separated by a growth rate threshold, *r*_mf_ (the growth rate of multi-fork replication). HU treatment, known to induce DNA replication fork stalling, increases the cell length variability of only those cells which are undergoing rapid growth. This HU-induced variability is further enhanced in a ΔrecA mutant when compared with wild-type. The linear increase in RecA co-localization on the nucleoid with growth rate and cell length variability suggests the involvement of the RecA protein in coupling increasing growth rates to higher cell length variability.

In general, stochastic partitioning of subcellular components has been shown to be a major source of cell phenotypic variability in a study combining theory and experiment [23]. Our observations on the role of stochastic replication dynamics potentially add to the potential contributors to phenotypic variability or ‘noise’. We expect stochastic DNA replication–segregation effects on cell size, should result in cell elongation owing to incomplete segregation of DNA. In agar-pad growth experiments in DIC and fluorescence of *E. coli* MG1655 with nucleoids labelled by HupA-GFP, most cells divide to produce newborn cells with typical birth lengths of approximately 2 µm after successfully segregating their nucleoids in approximately 20 min (electronic supplementary material, figure S4*a* and video S1). On the other hand, rare cells become approximately 40 µm long, after their nucleoids fail to segregate even after 50 min (electronic supplementary material, figure S4*b* and video S1). Growing wild-type and mutant strains in multiple growth media with HU and trimethoprim, we show that cell length variability is greater when replication processivity is perturbed, depending on the growth rate.

Cell size is a complex phenotype and is influenced by multiple pathways such as nutrient sensing [9–11,59], the division site selection by the minCDE proteins [60,61], nucleoid occlusion to sense incomplete replication [51,62] and the SOS response pathway [34,35]. However, this is the first study, to the best of our knowledge, that proposes a mechanism connecting growth rate with cell length variability based on multi-fork replication. It remains to be seen if a more direct method of replication fork tracking [30] can be used to test this proposed mechanism.

In previous studies on mammalian cell size regulation, the statistics of fixed cells were used to estimate the ‘variability’ [63]. While many older studies on bacterial cell length regulation [2,64] made use of such an approach, the advent of single-cell approaches have improved the robustness and accuracy of *E. coli* cell size and division measurements [18,19]. However, in the process, the population effects have been ignored. In this work, we attempt to bridge this gap. Additionally, we test our method of population variability measurement for artefacts that could result from a lack of cell cycle stage synchronization of the population. Indeed by measuring the growth of populations under different growth conditions, we find that the growth rate dependence of cell size variability is related to genome copy numbers per cell and is independent of synchronization.

We find the genome-copy number per cell, driven by growth rate, appears to positively correlate with cell size distributions in the population. An analogous study in yeast has examined the effect of the ploidy of specific genes on cell size regulation [65]. In the case of this study, while the genome copies per cell increase cell size variability, we have not estimated the possible role of specific genes and their ploidy on cell sizes. Additionally, our microcolony analysis once more reveals growth rate differences between clonal individuals under identical conditions (figure 3*d*). While these differences are not addressed in our study, it would be useful to extend our current analysis to the possible role of specific genes and proteins in single-cell growth rate variability.

**Figure 7.**
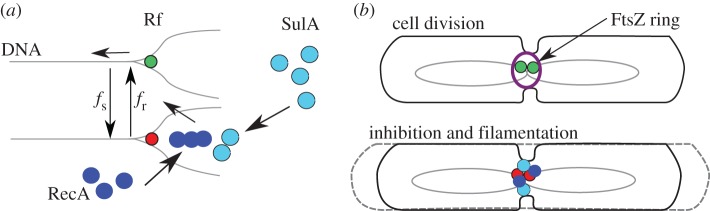
A proposed model of RecA recruitment and cell length regulation. (*a*) The stalled DNA-replication forks (red) result in the recruitment of RecA (dark blue) to the DNA and trigger SulA expression (light blue). The transition of recovered (green) and stalled replication forks is stochastic (determined by the frequency of stalling, *f*_s_) and reversible (determined by the frequency of recovery, *f*_r_). RecA filament assembly promotes increased recovery (*f*_r_). (*b*) In normal cell division, replication forks are in a recovered state and FtsZ assembles at the septum. Recruitment of RecA results in SulA-based inhibition of division and cell filamentation (long cell).

The RecA protein, examined in this study for a relationship with growth-rate-dependent cell size variability, is an SOS response pathway protein. It has previously been shown to enhance the recovery of stalled replication forks [29,66]. At the same time, when RecA is recruited to DNA, it activates SulA, which sequesters FtsZ monomers [34], thus acting as a cell division inhibitor. Fast-growing *E. coli* are also known to initiate multiple replication forks [16,67]. DNA replication fork progression is known to be stochastic [68]. The increase in cell length variability that we observe as a function of growth rate in wild-type *E. coli* can thus be explained by a model where RecA recruitment to stalled replication forks (figure 7*a*) leads to an increase in the proportion of elongated cells owing to cell-division inhibition (figure 7*b*). The cause for the onset of this process during rapid growth, we hypothesize, results from the probabilistic replication fork stalling (figure 7*a*) and the multiplicative effect owing to multi-fork replication [16]. While a computer simulation of multi-fork replication in the *E. coli* cell cycle exists [49], an explicit model of replication stochasticity coupled to multi-fork replication dynamics could help further test our hypothesis.

Our observations of the role of RecA in cell size variability have two parts: (i) the effect of a functional copy of *recA* and (ii) the effect of a *recA* deletion. (i) We observed population cell length variability of *E. coli* to increase with growth rate (figures 1–3) and tested the hypothesis that increasing RecA localization on the genome with increasing growth rate correlated with this growth rate dependence (figure 6). We find that RecA-SulA-mediated cell division inhibition increases cell length variability in rapid growth in wild-type cells (figure 4). Additionally, in rapidly growing cultures of the wild-type (LB, 37°C), overexpression of the RecA protein from the pRecA-mCherry (electronic supplementary material, figure S9*a*) and arabinose-inducible pBAD24-RecA constructs (electronic supplementary material, figure S9*b*) did not affect cell length variability (electronic supplementary material, figure S2*a,b*). In cells missing a copy of recA, treatment with 30 mM HU increased the variability of cell lengths (electronic supplementary material, figure S3*a*). The overexpression of plasmid-based copy of recA in these cells from pRecA-mCherry and pBAD24-recA plasmids reduced the spread of cell lengths to levels comparable to untreated MG1655 (electronic supplementary material, figure S3*b*). (ii) As reported previously, in the absence of RecA, the function of DNA replication fork stall rescue is hindered [31], resulting in replication defects [66] which are not repaired owing to the inability of the cell to induce an SOS response through LexA cleavage [69]. This results in cell division inhibition potentially owing to nucleoid occlusion [70,71] and additional RecA-independent pathways that detect incomplete replication [51]. However, distinct from previous work, we find greater variability in cell lengths in a *ΔrecA* strain when compared with hydroxyurea-treated wild-type cells in LB (figure 4*c*). We also demonstrate that the phenotype can be rescued by plasmid-based expression of the RecA protein, both with and without a fluorescent tag (figure 4*c* and electronic supplementary material, figure S2*a,b*). In future, our data could form the basis of a mathematical model, extending a previously developed model of *recA* gene expression dynamics during UV-based damage [72], focusing instead on the effect of growth rates.

HU treatment increases replication fork stalling and slows down DNA replication [73] and also results in cell division failure. Stalled replication forks that are not repaired, result in incomplete DNA replication [29] and result in elongated cells owing to cell division inhibition [12,13]. A direct measurement of replication fork dynamics of a population of dividing bacteria based on methods used to study the single molecule replication dynamics [30,74] could be used to potentially test our predictions at a subcellular level. Additionally, recently developed artificial ‘replication roadblocks’ in *E. coli* [30] and *Bacillus subtilis* [62] could be used in future to generate known number of replication fork stalling events and quantify their effect on population cell lengths, as a further test of the model.

The role of small proportions of outliers or ‘tails’ in phenotypic variability of a population has been shown to confer advantages to ‘persister’ cells, when the population undergoes selection [57,58,75]. However, a clear functional role for cell lengths is yet to be unambiguously determined. Suggestive evidence from clinical isolates of uropathogenic *E. coli* have implied that filamentous cells are harder for immune cells to clear than those of normal length [76]. In future, a study of the possible role of cell size and shape of not just *E. coli* in their natural environment could shed more light on the possible role in cell survival.

Our results suggest that an increased genome copy numbers in *E. coli* increases cell size heterogeneity. This is consistent with single-cell measurements in *E. coli* [30], but in contrast with *S. cerevisiae,* which shows that increased genome copy numbers lead to reduced ‘noise’ in cell size distributions [65]. To infer general principles from these results, the distribution of DNA replication origins in yeast and the concurrent nature of replication in bacteria will need to be taken into account. It can be presumed that the effect we report will only occur in organisms where rapid growth entails multiple simultaneous rounds of DNA replication.

## 5. Conclusion

In conclusion, we find that cell length variability of wild-type *E. coli* increases with increasing growth rate in a non-monotonic manner above a growth rate threshold for multi-fork replication (*r*_mf_). This variability is independent of cell-cycle stage synchronization of the population. Increasing HU concentrations to modulate replication stochasticity only changes the cell length distributions of populations undergoing rapid growth, an effect amplified in Δ*recA* cells. The rescue of increased cell length variability by RecA expression in deletion mutants, the effect of replication stalling induction on cell lengths and recruitment of RecA to the DNA, all indicate a model of stochastic multi-fork replication involving SOS response proteins. This could provide a mechanistic explanation of how growth rate affects population cell length variability in *E. coli*.

## Supplementary Material

Supplementary Materials

## Data accessibility

The software code developed as a part of this manuscript has been released as Open Source software and can be freely downloaded from https://github.com/athale/ecolilenDIC. The link to the program is also available from the Dryad Digital Repository: http://dx.doi.org/10.5061/dryad.2bs69. All experimental data are included in this manuscript and the accompanying electronic supplementary materials [77].
