# Supplementary material for: Threshold effect of growth rate on population variability of Escherichia coli cell lengths
Source: R Soc Open Sci. 2017 Feb 22;4(2):160417. doi: 10.1098/rsos.160417 (PMC5367290; doi:10.1098/rsos.160417)
Supplement: Supplementary Materials [file rsos160417supp1.docx]

**Supplementary Materials**

**Threshold Effect of Growth Rate on Population Variability of *E. coli* Cell Lengths**

Manasi S. Gangan and Chaitanya A. Athale.

**Contents:**

**1. Supplementary Tables**

**2. Supplementary Figures**

**3. Supplementary Videos**

**1. Supplementary Tables**

**Table S1.** Primers used to amplify the *E. coli* MG1655 genomic copy of *recA*, with overhanging regions containing restriction sites cloning into p-mCherry and pBAD24 plasmids. The *recA* genes were amplified from *E. coli* MG1655 genomic DNA.

| **Primer** | **Orientation** | **Restriction site** | **Sequence (5’- 3’)** |
| --- | --- | --- | --- |
| RecA-mcherry-f | Forward | HindIII | GCT AAG CTT ATG GCT ATC GAC GAA AA |
| RecA-mcherry-r | Reverse | SalI | GCA GTC GAC ATA AAA TCT TCG TTA GTT TCT |
| BAD24-recA-f | Forward | NheI | AAA GCT AGC ATG GCT ATC GAC GAA AA |
| BAD24-recA-r | Reverse | XbaI | GCC TCT AGA TTA AAA ATC TTC GTT AGT TT |

**Table S2.** The O.D. of the cultures harvested for Western blotting (Fig. S8) in LB and reduced-media (YEB, TB) before and after hydroxyurea (HU) treatment.

| *Growth medium* | *OD at 600nm* | |
| --- | --- | --- |
|  | *Pre-treatment* | *Recovery after HU treatment* |
| LB | 0.114 | 0.915 |
| YEB | 0.156 | 1.201 |
| TB | 0.185 | 1.130 |

**Table S3.** The values Kolmogorov-Smirnov test statistic (D_(α,n)_) used to test the goodness of fit of lognormal distributions from different cultures and growth conditions.

| **Growth conditions** | **K-S test statistic values** |
| --- | --- |
| **Media (log phase cultures)** | **df = 43 and α = 0.001** |
| LB | d_max_ = 24.9  D = 68.0 |
| Yeast extract broth (YEB) | d_max_ = 59.97  D = 86.9 |
| Tryptone broth (TB) | d_max_ = 63.1  D = 68.26 |
| M9+Glucose | d_max_ = 24.8  D = 44.0 |
| M9+Succinate | d_max_ = 108.2  D = 118.0 |
| M9+Acetate | d_max_ = 7.5  D = 32.7 |
| **‘Mother machine’ cell cycle phase** | **df = 43 and α = 0.001** |
| Birth | d_max_ = 6.29  D = 24.11 |
| Division | d_max_ = 6.23  D = 20.45 |
| Birth (M9 + succinate) | d_max_ = 5.15  D = 22.48 |
| Division (M9 + succinate) | d_max_ = 0.611  D = 16.08 |
| **Mid-log phase fit to lognormal for different strains** | **df = 43 and α = 0.001** |
| *E. coli* MG1655 | d_max_= 37.55  D = 70.34 |
| *E. coli ∆recA* | d_max_ = 28.82  D = 75.0 |
| *E. coli ∆sulA* | d_max_ = 60.39  D = 72.4 |
| *E. coli* MG1655 + pRecA- mCherry | d_max_ = 10.36  D = 22.14 |
| *E. coli ∆recA* + pRecA- mCherry | d_max_ = 46.59  D = 68.23 |
| *E. coli* MG1655 + pBAD24-RecA | d_max_ = 28.89  D = 32.33 |
| *E. coli ∆recA* + pBAD24-RecA | d_max_ = 36.37  D = 97.61 |

**2. Supplementary Figures**

**Fig. S1:** The experimentally measured and averaged (n=3) O.D. as a function of time data (black dots) was fit by the logistic equation (red) to obtain the growth rate (r) and carrying capacity (K) of cultures grown in LB, YE, TB and M9 with sugars. The R^2^ value indicates the goodness of fit.

**Fig. S2:** Cell length variability in SOS response mutants. (A) The cell length frequency distribution of ~10^3^ cells (bars) from mid- log phase cultures in LB of the following *E. coli* strains were fit to a lognormal distribution (red): MG1655, *∆recA*, *∆sulA*, MG11655 + pRecA-mCherry, *∆recA* + pRecA-mCherry, MG11655 + pBAD24-RecA (+ 0.7% arabinose) and *∆recA*+pBAD24-RecA (+ 0.7% arabinose). (B) The coefficient of variation (CV) of cell lengths (y-axis) was plotted for the same *E. coli* strains based on (A).

**Fig. S3:** RecA complementation in the presence of hydroxyurea. (A) The cell length frequency distribution of ~10^3^ cells (bars) sampled from the mid-log phase of LB grown cultures are compared between untreated MG1655 and 30 mM hydroxurea (HU) treated cells of MG1655. The cell length distribution of ∆recA cells treated with 30 mM HU is contrasted with the same background with pRecA-mCherry and pBAD24-RecA (+ 0.7% arabinose). All distributions were fit to a lognormal distribution (red). (B) The cell length variability estimated by CV is plotted for the *E. coli* strains described in (A).

**Fig. S4**: Cell size correlates with nucleoid segregation. (A) The division of an MG1655 cell is followed (26 min) and the long-axis of the same cell (arrow) is used to generate a kymograph of HupA-GFP labeled nucleoid segregation. (B) The growth of a filamentous MG1655 cell (arrow) is correlated with the failed segregation of nucleoids seen in the nucleoid kymography (Scale bar: 5 μm).

**Fig. S5:** (A) *E. coli* *ΔrecA* cells were transformed with a pBAD24-RecA construct and cultured in LB+0.7% (w/v) arabinose or M9+0.7% (w/v) arabinose. The fixed cell images in DIC compare samples without and with hydroxyurea (HU) treatment. (B) The cell length variability is compared between cells that were untreated (black) and treated with 30 mM HU (red), compared between growth in LB+Arabinose (0.7%) and M9+Arabinose (0.7%).

**Fig. S6:** Immuno-localization patterns of RecA in *E. coli* MG1655. (A) Images were acquired in three regions of interest (ROI1, 2 and 3) of *E. coli* MG1655 cells in DIC (grey) with the nucleoid stained by DAPI (red) and RecA stained by anti-recA serum as primary and Alexa fluor-647 tagged anti-Rabbit antibodies as secondary (green). The ROIs are marked ROI 1, ROI 2 and ROI 3. (B) The percentage of cells in which the nucleoid stain colocalized with RecA-GFP (black) and the anti-RecA antibody (red) is plotted. (C) The coefficient of variation of cell lengths (y-axis) of *E. coli* cells with an endogenous RecA-GFP (black) is compared to that of MG1655 cells stained with anti-RecA serum (red).

**Fig. S7:** Localization of RecA-mCherry in *E. coli ∆recA*. (A) Fixed cells of *E. coli ∆recA* transformed with the *pRecA-mCherry* plasmid are imaged in DIC (grey), DAPI (red) and mCherry (green) channels. The merge of DAPI and RecA-mCherry channels indicates co-localization (yellow). Scale bar- 5 µm. (B) The percentage of cells with RecA co-localization with the DAPI stained nucleoid and (C) cell length variability (CV_L_) are compared between *E. coli MG1655* stained by anti-RecA antibodies (black), *E. coli MG1655-recA-GFP* (red bar) and *E. coli ∆recA* with the *pRecA-mCherry* plasmid (blue).

**Fig. S8:** Anti-RecA antiserum (43) was used to detect RecA protein levels in the cells using a western blot. (A) An immunoblot of the cell lysates of *E. coli* MG1655 grown in LB (1x and 2x lysate), TB and YEB are compared between untreated and 30 mM hydroxyurea (HU) treated samples. The yellow box marks the *E. coli* RecA (37,890 Da) band. Additionally, cell lysates of *E. coli* MG1655-*recA-GFP*, *E. coli ΔrecA* with the *pRecA-mCherry* plasmid and *E. coli ΔrecA* with the *pBAD24-RecA* plasmid were also detected. The RecA-GFP (green) and RecA-mCherry (red) fusion protein bands are highlighted. (B) The band-intensity of RecA is plotted for LB, YEB and TB with and without treatment. The 2X samples are two-fold higher cell mass equivalents loaded as controls for quantification. The bar-graphs are normalized by the maximum. (C) A Coomassie Brilliant Blue (CBB) stained gel of the same samples as in (A) was analyzed for band intensity using three visible bands (black, yellow and green) and (D) the mean intensity averaged (±s.d.) for the three bands plotted for each sample as a loading control. (E) An immunoblot for RecA (marked by a yellow box) was used to estimate (F) intensity differences between RecA-GFP expression in *E. coli* *MG1655-recA-GFP* cells- untreated and treated with 30 mM hydroxurea. (G) A CBB stained gel of the same was analyzed for the band intensity of three non-specific bands (black, yellow and green). (H) The mean (±s.d.) intensity of the three bands from the samples in (E) is plotted.

**Fig. S9:** The RecA plasmid constructs. (A) The *pRecA-mCherry* plasmid with *recA-mCherry* expressed under the control of a lactose-inducible promoter. (B) The *pBAD24-recA* plasmid with *recA* expression controlled by an arabinose-inducible promoter.

**Fig. S1.**

**Fig. S2.**


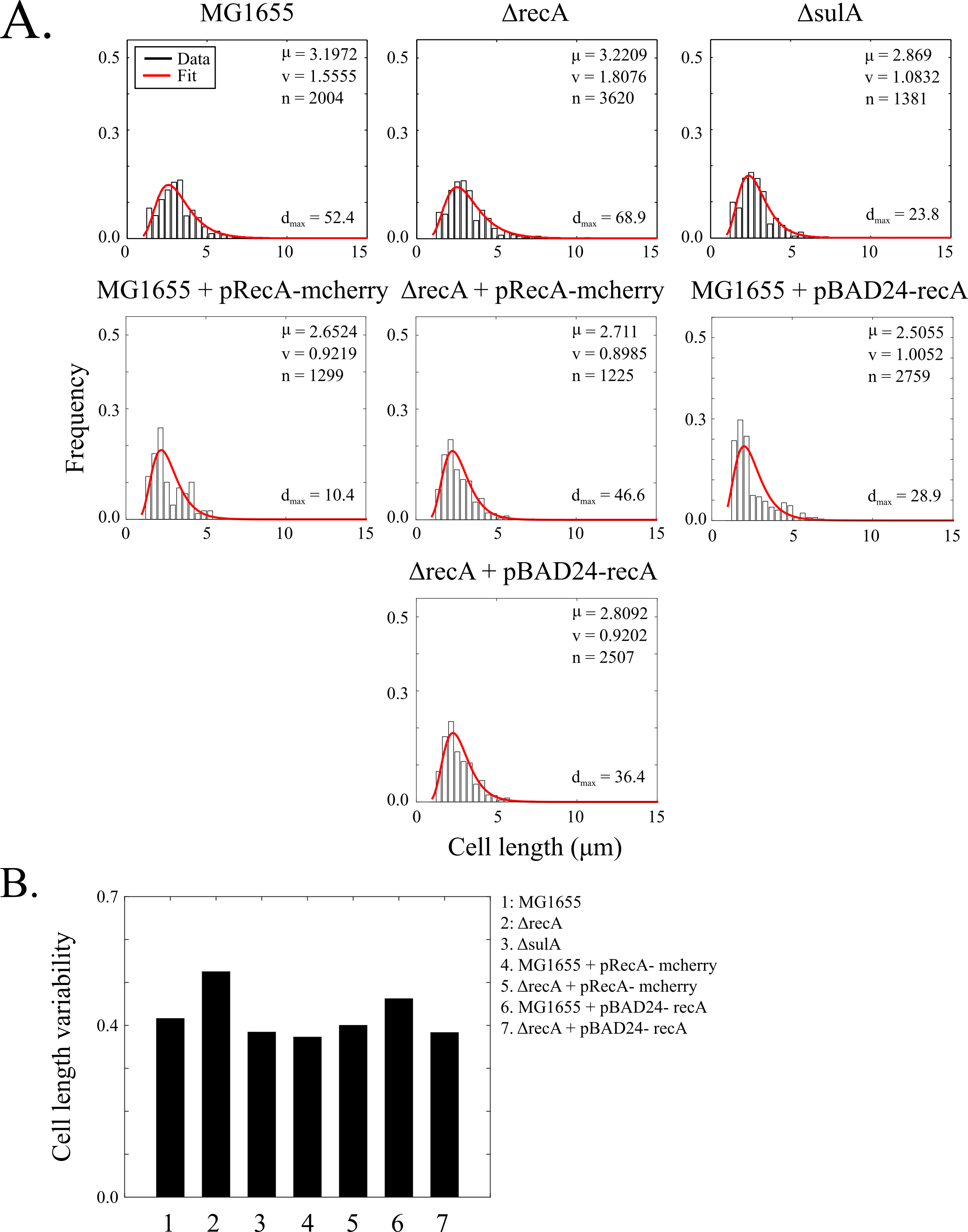


**Fig. S3.**

**Fig. S4**.

**Fig. S5**.


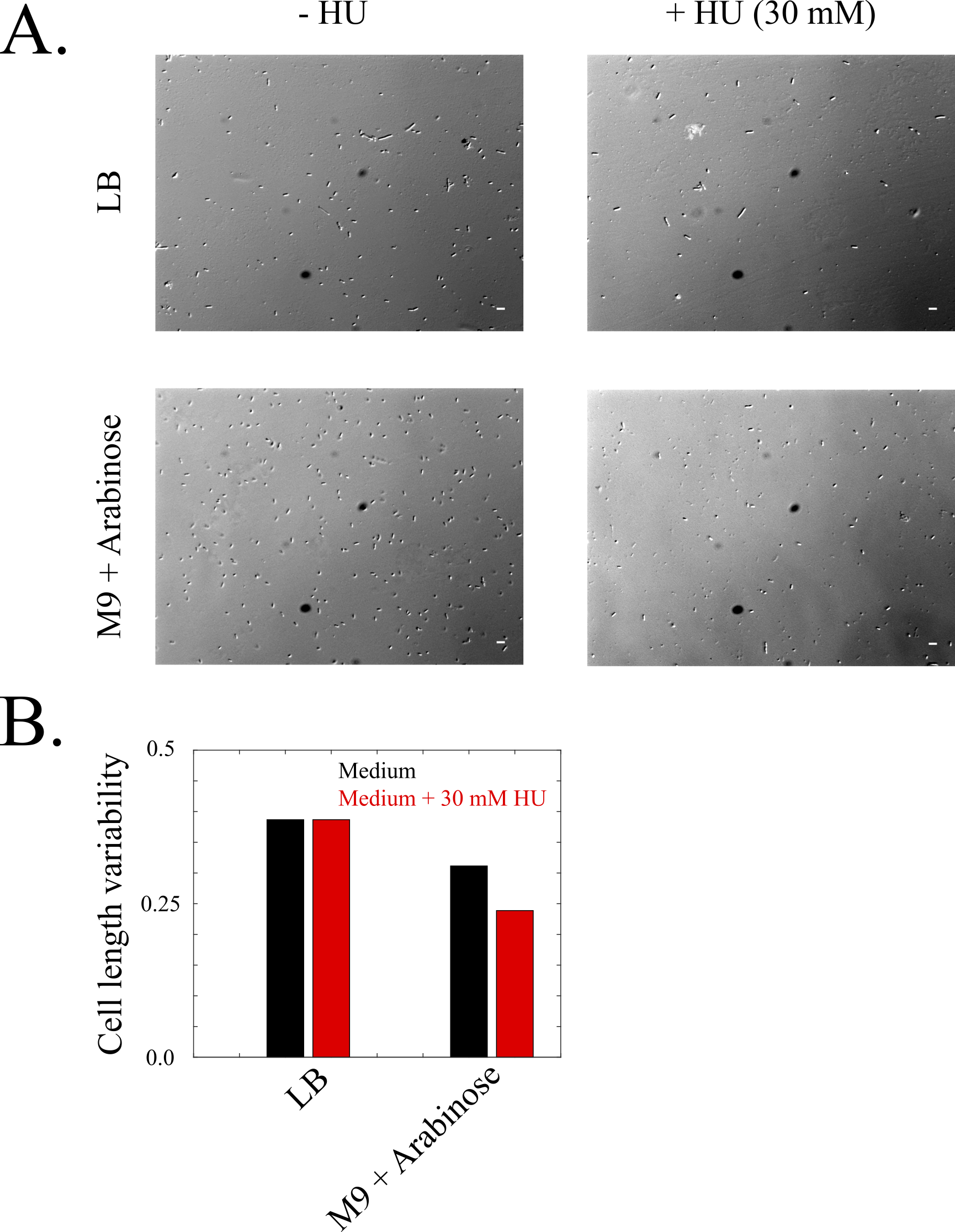


**Fig. S6**.

**
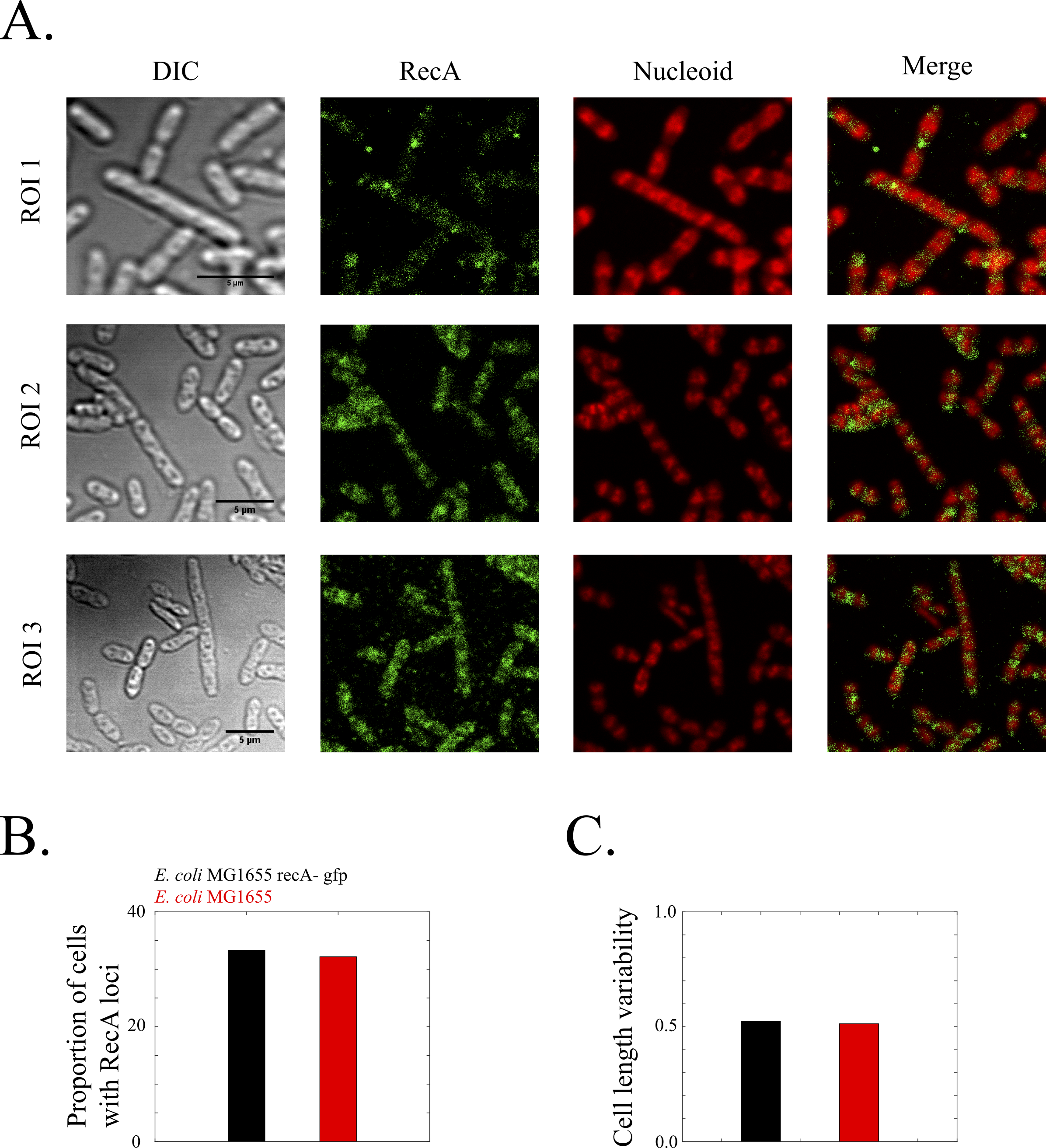
**

**Fig. S7.**

**
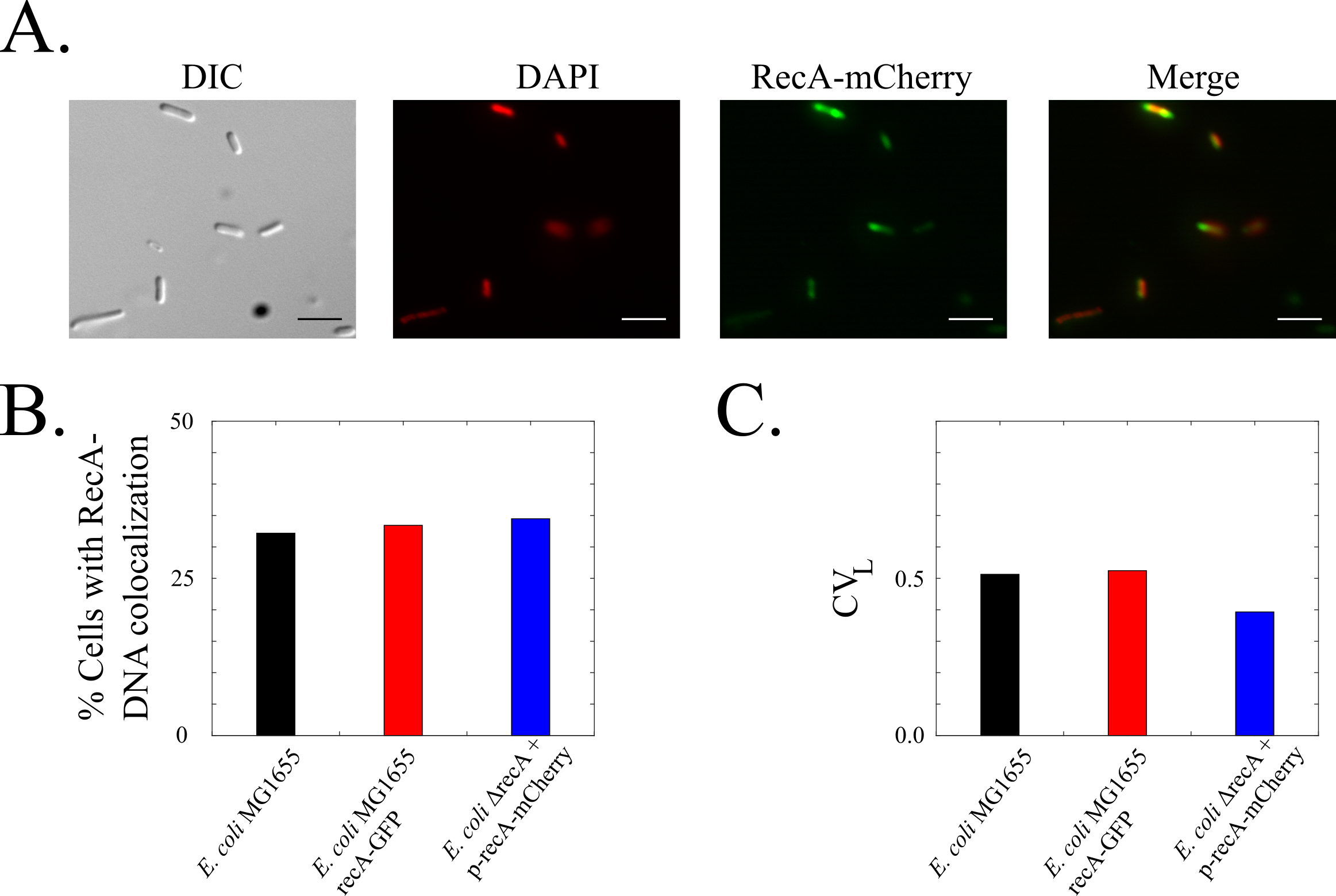
**

**Fig. S8.**

**Fig. S9.**

**
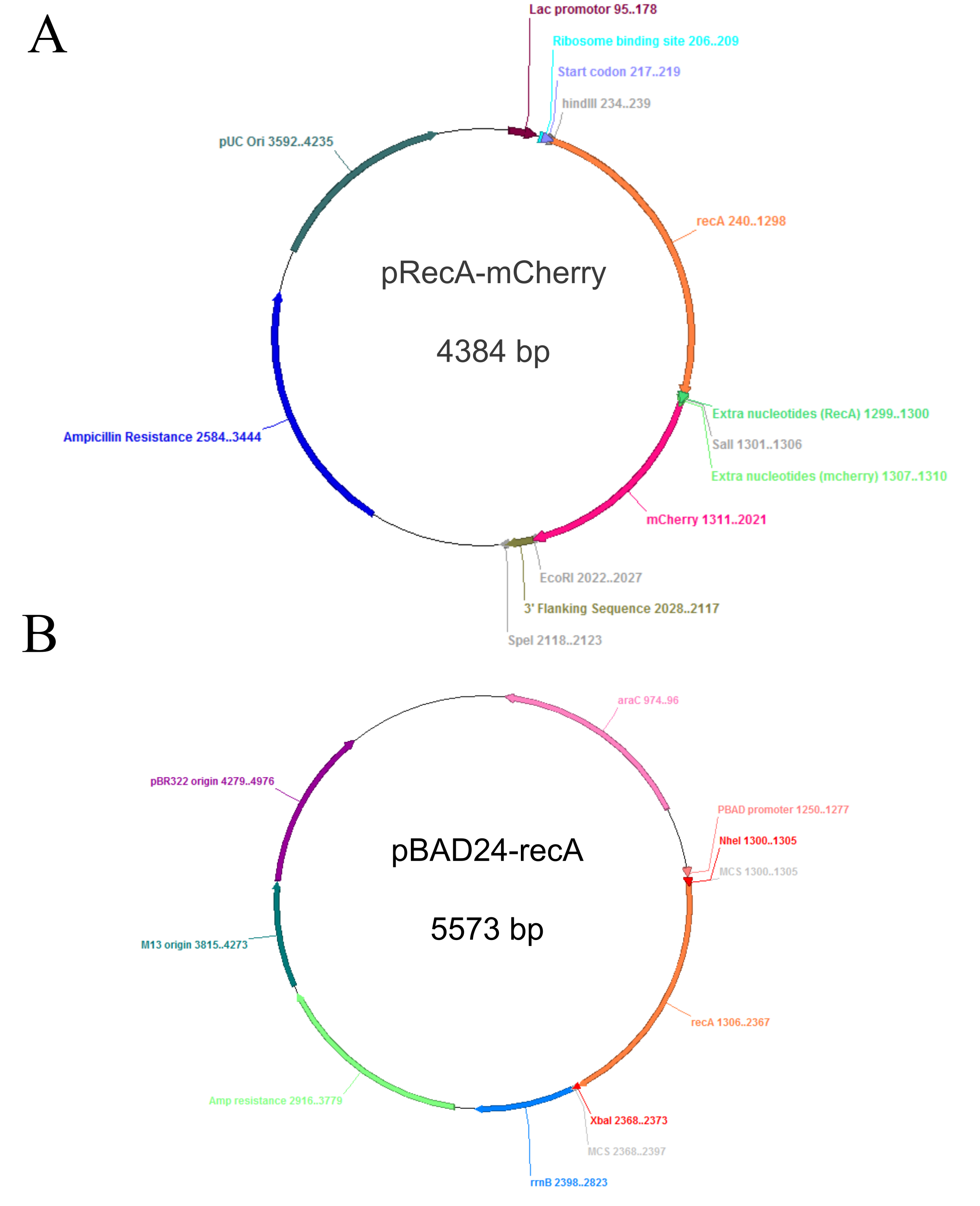
**

**3. Supplementary Videos**

**Video S1:** The growth and division of a population of *E. coli* MG1655 cells on an agar-pad is followed in DIC (green) simultaneously with the HupA-GFP (red). The time-stamp is in units hh:mm. The arrow indicates a cell elongation event corresponding to a delay in nucleoid segregation. The scale bar is 10 µm.

**Video S2:** Time-lapse images of continuous culture of *E. coli* MG1655 cells grown in LB were acquired in DIC microscopy. The channels restricting the movement of the cells correspond to the dead-end channels of the ‘mother machine’, while the central trench corresponds to the nutrient channel. The interval between each frame is 100 seconds. The time stamp is hh:mm:ss. Scale bar: 5 µm.

**Video S3:** Time-lapse images of *E. coli* MG1655 cells in continuous culture grown in the ‘mother machine’ with M9 + succinate as the medium at 37°C were recorded in DIC. Images were acquired every 120 s. The time stamp is hh:mm:ss. Scale bar: 5 µm.

**Video S4:** DIC time series of E. coli MG1655 micro-colony originated from single mother cell and growing on agar pad at 37°C was imaged at an interval of 2 minutes between every frame. The time stamp is hh:mm:ss (Scale bar: 5 µm).

**Video S5:** *E. coli* MG1655 cells expressing eGFP were grown in the ‘mother machine’ in LB at 37°C for 3 hours and fluorescence images in the GFP channel acquired every 2 minutes. The time stamp is hh:mm:ss. Scale bar: 5 µm.

**Video S6:** *E. coli* MG1655 cells expressing eGFP were grown in the ‘mother machine’ with a continuous flow of LB for 1 hour. The medium was changed to LB + 30 mM hydroxyurea for 1 hour of ‘treatment’. The medium was changed to LB alone for 2.5 hours for ‘recovery’. Fluorescence time-lapse images were acquired in the GFP channel every 2 minutes. The time stamp is hh:mm:ss. Scale bar: 5 µm.

**Video S7:** *E. coli* ∆recA cells expressing eGFP were grown in LB at 37°C for 3 hours in the ‘mother machine’ with a continuous flow of LB. Fluorescence time lapse images were acquired every 2 minutes in the GFP channel. Time stamp is hh:mm:ss. Scale bar: 5 µm.

**Video S8:** *E. coli* ∆recA cells expressing plasmid-based eGFP were grown in the ‘mother machine’ with LB for 1 hour, followed by LB+hydroxyurea containing medium for 1 hour (‘treatment’) and LB alone for 2.5 hours (‘recovery’). Fluorescence images were acquired every 2 minutes in the GFP channel. The time stamp is hh:mm:ss. Scale bar: 5 µm
